# Consilient Care: When Physical and Mental Health Issues Coexist

**DOI:** 10.7759/cureus.85335

**Published:** 2025-06-04

**Authors:** Tyler C Cymet

**Affiliations:** 1 Medicine and Primary Care, Illinois College of Osteopathic Medicine at The Chicago School, Chicago, USA

**Keywords:** consilient care, diagnostic overshadowing, physical symptoms, psychological issues, somatization disorder

## Abstract

The intricate relationship between physical and mental health presents a significant challenge in modern healthcare. This editorial introduces the concept of *consilient care, *an integrated, interdisciplinary approach that bridges mental and physical health services to improve patient outcomes. It explores the bidirectional impact of mental disorders on physical health and vice versa, emphasizing the need for holistic, patient-centered strategies. The discussion spans emergency and internal medicine, highlighting the diagnostic complexities of overlapping symptoms and the importance of standardized assessment tools. It also outlines practical interventions, including integrated care models, lifestyle modifications, medication management, and patient education. Drawing on insights from the World Psychiatric Association and the World Health Organization, the editorial advocates for systemic reforms that prioritize comprehensive care for individuals facing coexisting mental and physical health challenges.

## Editorial

Managing physical health in mental illness and mental health in physical illness: a consilient approach

The relationship between physical and mental health is deeply intertwined. Individuals with mental disorders often struggle with physical health challenges, while those with physical illnesses frequently experience psychological distress. This editorial explores strategies for managing physical health in people with mental disorders and mental health in people with physical disorders.

The case for consilient care

Consilient care refers to the integration of mental and physical health to improve patient outcomes. Rather than treating these domains in isolation, consilient care promotes a collaborative, interdisciplinary model that recognizes the interconnected nature of health. By uniting these disciplines, consilient care fosters a holistic approach, leveraging the strengths and insights of each field to create comprehensive, patient-centered solutions. This collaborative model enhances innovation, efficiency, and effectiveness in healthcare delivery, ultimately leading to better health outcomes and patient satisfaction. Integrating mental and physical health is crucial because it addresses the interconnected nature of these aspects of health, reducing stigma and improving overall well-being [1].

Overlapping fields of medicine: emergency medicine and internal medicine

In emergency medicine, the overlap between physical and mental health is especially pronounced. Patients often present with symptoms that defy simple categorization: chronic pain, somatic syndromes, and functional neurological disorders like psychogenic nonepileptic seizures are just a few examples. Chronic pain is a classic example of a symptom that is hard to classify into just one category. These conditions blur the lines between physical and psychological origins, complicating diagnosis and treatment.

To navigate this complexity, emergency providers must rely on standardized tools such as the visual analog scale for pain, the SF-36 for quality of life, and mental health screeners like the GAD-7 and PHQ-9. These instruments help ensure consistent communication and longitudinal tracking of symptoms [2].

Similarly, in internal medicine, chronic illnesses such as lupus, rheumatoid arthritis, and irritable bowel syndrome are often exacerbated by stress and emotional distress. Cardiovascular disease, too, is frequently accompanied by anxiety and depression. These examples underscore the need for integrated care that addresses both the physical and psychological dimensions of illness.

Supporting physical health for people with mental disorders 

People with severe mental illnesses, such as schizophrenia, bipolar disorder, and major depression, face elevated risks of physical health problems, including cardiovascular disease and diabetes. These individuals often experience premature mortality due to preventable conditions, compounded by barriers to care and self-management challenges.

Symptom shadowing

In clinical practice, more visible or urgent symptoms (e.g., high blood glucose, weight gain) often overshadow core psychological issues like depression. This can lead to underdiagnosis or undertreatment of mental health conditions in patients with chronic illnesses.

Integrated care models

Integrated care models that combine mental and physical health services are essential for improving health outcomes in people with severe mental disorders. This takes a team, a personalized care plan, and ongoing monitoring and support. Best practices for this type of care have not been successfully implemented yet, and siloed training makes it harder for any one provider to develop comprehensive treatment plans. A new model that can facilitate coordinated care is needed, ensuring that both mental and physical health conditions are addressed simultaneously. This is especially important in chronic conditions where overlap in the source of ill health will eventually occur, comorbid conditions, and when there are issues related to accessibility and affordability of care. 

Bringing pharmaceutical interventions into this model with the significant physical side effects that psychotropic medications have like weight gain and metabolic syndrome further complicate creation of new systems of integrated care.

Education and lifestyle support

Educating individuals with mental disorders about the importance of physical health and providing support for healthy lifestyle choices can empower them to take an active role in their health management. How healthcare interventions transition from the exam room to the real world and are put into practice is often referred to as the 46th minute since the interface in therapy sessions is historically 45 minutes long. How people will process, reflect and apply the healthcare interventions prescribed or suggested and how a therapy session extends from the exam room to the patient's living room require a broad focus that integrates physical health as well.

Mental health management for people with physical disorders

Physical disorders, particularly chronic conditions such as cardiovascular disease, diabetes, and cancer, can have a profound impact on mental health. The stress and challenges associated with managing a physical illness can lead to depression, anxiety, and other mental health issues. Effective management of mental health in people with physical disorders requires a holistic approach that addresses both physical and psychological needs.

Psychological interventions, such as cognitive-behavioral therapy and mindfulness-based stress reduction, can help individuals cope with the emotional burden of physical illness. These therapies can reduce symptoms of depression and anxiety and improve overall well-being.

Peer support and community

Support groups provide a platform for individuals with physical disorders to share their experiences and receive emotional support. These groups can reduce feelings of isolation and promote mental health.

Like integrated care models similar to the management of physical health in people with mental disorders, integrated care models that combine physical and mental health services are also beneficial. These models ensure that mental health needs are addressed alongside physical health management.

Educating patients about the potential mental health impacts of their physical conditions and providing resources for mental health support can help them manage their emotional well-being.

Insights from the World Psychiatric Association and World Health Organization workgroups

The World Psychiatric Association (WPA) and World Health Organization (WHO) have both recognized the importance of addressing comorbidities between mental and physical health. Their workgroups have developed guidelines and recommendations to improve the management of these comorbidities [3].

WPA workgroup on comorbidity

The WPA workgroup on comorbidity focuses on identifying topics related to the comorbidity of mental and physical disorders and developing recommendations for research, education, and service development. Their efforts include organizing symposia, workshops, and training programs to build capacity in managing comorbidities.

WHO guidelines

The WHO has published guidelines on the management of physical health conditions in adults with severe mental disorders. These guidelines provide evidence-based recommendations for healthcare providers on how to recognize and manage comorbid physical and mental health conditions.

Collaborative efforts from both the WPA and WHO emphasize the importance of collaborative efforts between mental health and physical health professionals. By working together, these professionals can provide comprehensive care that addresses the full spectrum of health needs [4].

Conclusion

Managing the physical health of people with mental disorders and the mental health of people with physical disorders requires a holistic and integrated approach. By combining physical and mental health services, providing education and support, and implementing evidence-based interventions, healthcare providers can improve health outcomes for these populations. The work of the WPA and WHO workgroups on comorbidities highlights the importance of addressing the complex interplay between mental and physical health and provides valuable guidelines for effective management.
